# Targeting glycosylation of PD-1 to enhance CAR-T cell cytotoxicity

**DOI:** 10.1186/s13045-019-0831-5

**Published:** 2019-11-29

**Authors:** Xiaojuan Shi, Daiqun Zhang, Feng Li, Zhen Zhang, Shumin Wang, Yujing Xuan, Yu Ping, Yi Zhang

**Affiliations:** 1 Biotherapy Center, The First Affiliated Hospital of Zhengzhou University, Zhengzhou, 450052 Henan China; 2 Cancer Center, The First Affiliated Hospital of Zhengzhou University, Zhengzhou, 450052 Henan China; 3 School of Life Sciences, Zhengzhou University, Zhengzhou, 450052 Henan China; 4 Henan Key Laboratory for Tumor Immunology and Biotherapy, Zhengzhou, 450052 Henan China

**Keywords:** Glycosylation, Single base editing, CAR-T cell, PD-1

## Abstract

Asparagine-linked (*N*-linked) glycosylation is ubiquitous and can stabilize immune inhibitory PD-1 protein. Reducing *N*-linked glycosylation of PD-1 may decrease PD-1 expression and relieve its inhibitory effects on CAR-T cells. Considering that the codon of Asparagine is aac or aat, we wondered if the adenine base editor (ABE), which induces a·t to g·c conversion at specific site, could be used to reduce PD-1 suppression by changing the glycosylated residue in CAR-T cells. Our results showed ABE editing altered the coding sequence of N74 residue of *PDCD1* and downregulated PD-1 expression in CAR-T cells. Further analysis showed ABE-edited CAR-T cells had enhanced cytotoxic functions in vitro and in vivo. Our study suggested that the single base editors can be used to augment CAR-T cell therapy.

To the Editor,

Chimeric antigen receptor T (CAR-T) cells are not satisfying in treating solid tumors [1]. PD-1 limits CAR-T cell therapy within solid tumors. CRISPR/cas9 can downregulate PD-1 [2] but also potentially leads to carcinogenesis, because success of such tools relies on suppressing DNA damage response [3]. Furthermore, CRISPR/cas9 could bring in missense mutations that might exacerbate T cell dysfunction. Hence, we need safer and more precise gene-editing tools to produce better CAR-T cells. *N*-linked glycosylation can stabilize PD-1 to compromise anti-tumor immunity [4]. As *N*-linked glycosylation is restricted on asparagine coded by aac or aat, adenine base editors (ABE) can convert a·t to g·c base pair [5], and may be used to diminish such glycosylation. Herein, we explored the potentials of ABE to edit and downregulate PD-1 in CAR-T cells.

Mutated PD-1 at N74 had decreased surface expression (Fig. 1a). Therefore, N74 in PD-1 is a good target for ABE. Three types of amino acids may be produced after base editing at N74 coded by aac (Fig. 1b). All 3 types of mutations into D74 (gac), S74 (agc), and G74 (ggc) comparably downregulated the surface and total PD-1 (*P* < 0.001) (Fig. 1c and Additional file 1: Figure S1c). Next, we investigated whether ABE was able to decrease PD-1 in CAR-T cells. The delivery of gRNA using lentivirus is efficient [6], so we constructed lentiviral vectors simultaneously expressing mesothelin-directed CAR and gRNA targeting non-specific sites (scramble) or N74 of *PDCD1* (gRNA), under 2 independent promoters (Additional file 1: Figure S1a). T cell transduction efficacies were over 85% (Additional file 1: Figure S1b). Then the commercially synthesized ABE proteins were delivered into CAR-T cells by electroporation. Sequencing data showed the conversion to g majorly happened from the first adenine within N74 codon of *PDCD1* in CAR-T cells expressing specific gRNA (Fig. 1d). Conversion was also noticed at the second adenine with lower frequencies (Fig. 1d). This editing pattern is consistent with previous report [7].

**Fig. 1:**
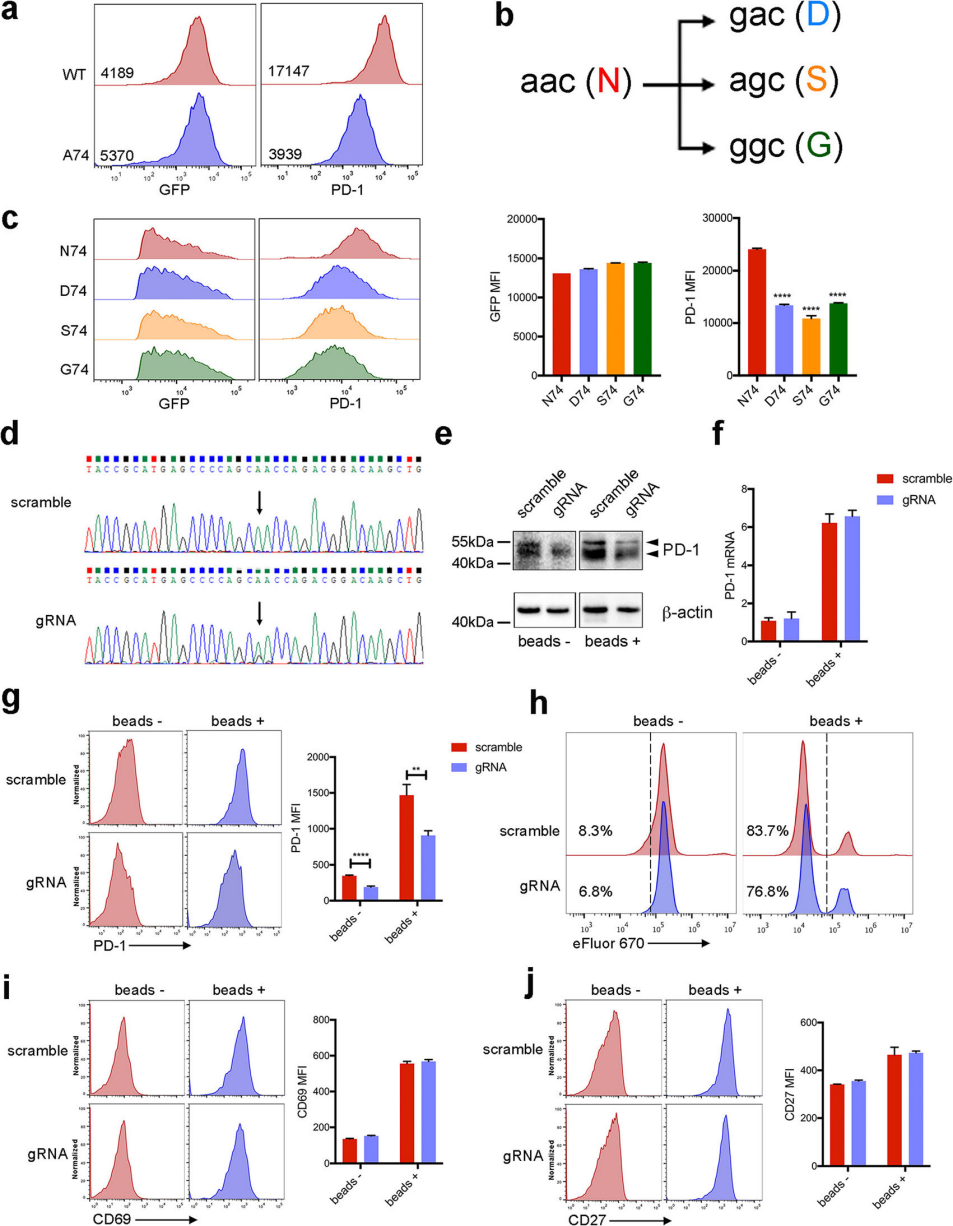
Mutations of N74 decreased PD-1. **a** Surface expressions of wild-type PD-1 and its derivate N74A (A74) mutation in 293 T cells. **b** Potential mutations resulted from single-nucleotide conversions at N74. **c** Mutations at N74 decreased surface expression of PD-1. PD-1 harboring wild-type or mutated N74 were tandemly linked with self-cleaving P2A and GFP, then transiently expressed in 293 T cells. Surface PD-1 expression was determined in GFP^+^ cells by FACS assay. **d** Sanger sequencing of *PDCD1* of CAR-T cells expressing scramble or N74-targeted gRNA after base editing. **e–j** CAR-T cells having comparable rates of GFP^+^ cells were activated with equal amounts of anti-CD3/CD28 beads without exogenous cytokines. **e** Western blots of PD-1 in CAR-T cells activated or not. **f** qRT-PCR detecting PD-1 expressions in resting and activated CAR-T cells. **g** Surface expressions of PD-1 in CAR-T cells before and after activation. And mean fluorescence intensity (MFI) values were compared. **h** CAR-T cells were stained with eFluor 670 dyes and then continued to culture with or without beads. Forty-eight hours later, proliferations of CAR-T cells were determined according to eFlour 670 dilution. Activation markers, CD69 (**i**) and CD27 (**j**) were detected and compared in different CAR-T cells before and after activation. **P < 0.01 and ****P < 0.001

In following experiments, the ratios of CAR-expressing cells were comparably adjusted to 85%. In gRNA CAR-T cells, PD-1 expression was decreased at protein level but not at mRNA level (Fig. 1e and f). Consistently, surface PD-1 was remarkably decreased in resting and activated gRNA CAR-T cells (*P* < 0.01) (Fig. 1g). Further analysis suggested that ABE editing did not impair the proliferation and activation of CAR-T cells (*P* > 0.05) (Fig. 1h–j) when PD-L1 was absent. Then mesothelin-positive cells with high PD-L1 expression were prepared (Fig. 2a). After washing out exogenous cytokines, CAR-T cells and target cells were co-incubated. Upon target cell engagement, CAR-T cells divided efficiently (Fig. 2b). Compared with gRNA counterparts, the proliferations of CAR-T cells expressing scramble RNA were significantly suppressed (*P* < 0.05) (Fig. 2b). gRNA CAR-T cells had enhanced cytolytic capacities (*P* < 0.05) and increased secretions of IL-2 and IFN-γ (*P* < 0.05) after activation by tumor cells (Fig. 2c and d). To further confirm the effectiveness of ABE in relieving T cell inhibition, we checked the anti-tumor functions of CAR-T cells in vivo. Consistently, CAR-T cells expressing N74-targeted gRNA attained greater expansion (*P* < 0.05) (Fig. 2e and Additional file 2: Figure S2). Decreased surface PD-1 (*P* < 0.01) and upregulated activation markers (CD69 and CD27) (*P* < 0.05) were noticed on gRNA CAR-T cells (Fig. 2f and g). gRNA CAR-T cells more efficiently delayed tumor growth and improved overall survival when compared with scramble counterparts (*P* < 0.05) (Fig. 2h–j) (Additional files 3 and 4).

**Fig. 2:**
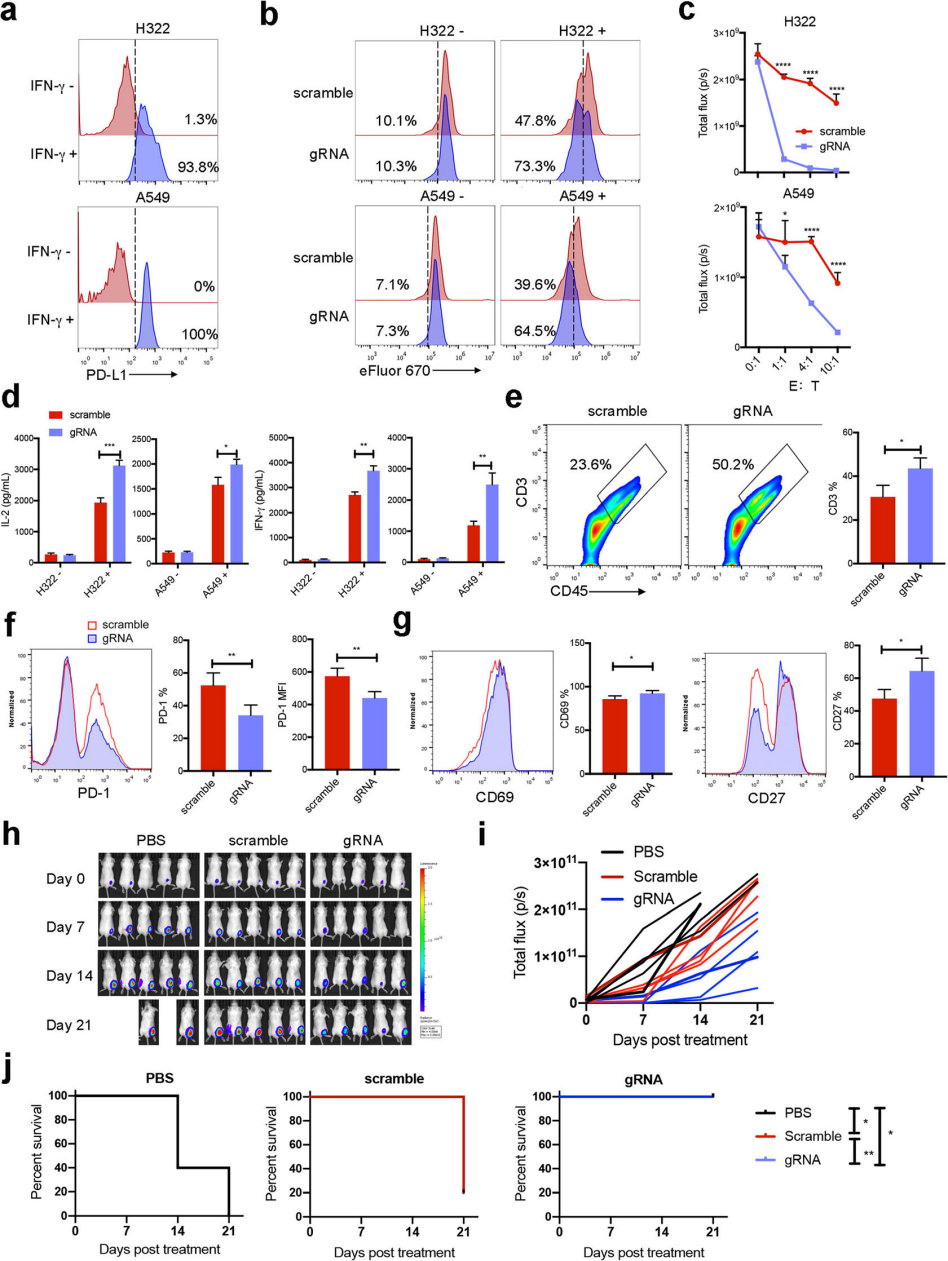
Single base conversion reduced PD-1-mediated suppression. **a** IFN-γ (100 IU/mL) induced PD-L1 expression in target cells. After that, target cells were washed to discard IFN-γ and used in following experiments. **b–d** CAR-T cells were co-incubated with target cells without exogenous cytokines. **b** CAR-T cells expanded with or without target cells for 48 h. **c** CAR-T cells were co-cultured with target cells at indicated effector to target ratios (E:T) for 24 h. The cytolytic potencies of CAR-T cells were tested using bioluminescence imaging. **d** CAR-T cells were incubated with tumor cells at E:T = 1:1. Twenty-four hour later, IL-2 and IFN-γ in supernatants were detected using ELISA. **e**–**j** The anti-tumor effects of ABE-edited CAR-T cells in vivo. **e** Five days after infusion, the ratios of infiltrated T cells (CD45^+^CD3^+^) were determined using flow cytometry after excluding dead cells (*n* = 4 per group). **f**, **g** The expressions of PD-1, CD69, and CD27 were detected in infiltrated T cells. In addition, effects of CAR-T cells on tumor growth (**h, i**) and survival of mice (**j**) were monitored weekly (each group had 5 mice). *P < 0.05 and **P < 0.01

Single base editing can modulate the stability and function of target protein by changing a single residue [8]. Our work further uncovered the potential of such editing tool in T cells. Compared with CRISPR/cas9, ABE has narrower editing window and much less frequent off-target events [9], representing a safer and more precise approach for gene editing. ABE-mediated point mutation can downregulate the inhibitory PD-1, therefore providing an alternative approach to augment T cell immunotherapy.

## Supplementary information

**Additional file 1: Figure S1.** CAR-T cell construction**.** (**a**) Structure of lentiviral vector simultaneously delivering CAR and gRNA. (**b**) Transduction efficacy of T cells. Transduction efficacies were determined by GFP expression on day 5, before performing single base editing on the same day. (**c**) Vectors coding wild type (N74) or mutated (D74, S74 or G74) PD-1 were transiently transfected into 293 T cells. 48 hours later, cell lysis was subjected to western blot analysis. This assay showed the alterations at N74 of *PDCD1* decreased the expression of PD-1 protein.

**Additional file 2: Figure S2.** CAR-T cells divided within tumor. Almost all the T cells (CD45^+^CD3^+^) accumulating within tumors were CAR-T cells (GFP^+^). In the infused T cells, about 85% were GFP^+^. In the activated T cells within tumors, the ratios of GFP^+^ cells were over 97%, indicating CAR-T cells but not the non-engineered cells divide upon antigen engagement *in vivo*. Untransduced T cells were used as control.

**Additional file 3: Table S1.** Antibodies and materials list.

**Additional file 4:.** Detailed method information and procedures of experiments.

## Data Availability

All supporting data are included in the manuscript and supplemental files. Additional data are available upon reasonable request to corresponding author.

## Abbreviations

ABE: Adenine base editor
CAR: Chimeric antigen receptor
CRISPR: Clustered regularly interspaced short palindromic repeats
DSB: Double-stranded break
gRNA: Guide RNA
IFN-γ: Interferon-γ
IL-2: Interleukin-2
