# Supplementary figures and images for: Targeting glycosylation of PD-1 to enhance CAR-T cell cytotoxicity
Source: J Hematol Oncol. 2019 Nov 29;12:127. doi: 10.1186/s13045-019-0831-5 (PMC6884797; doi:10.1186/s13045-019-0831-5)

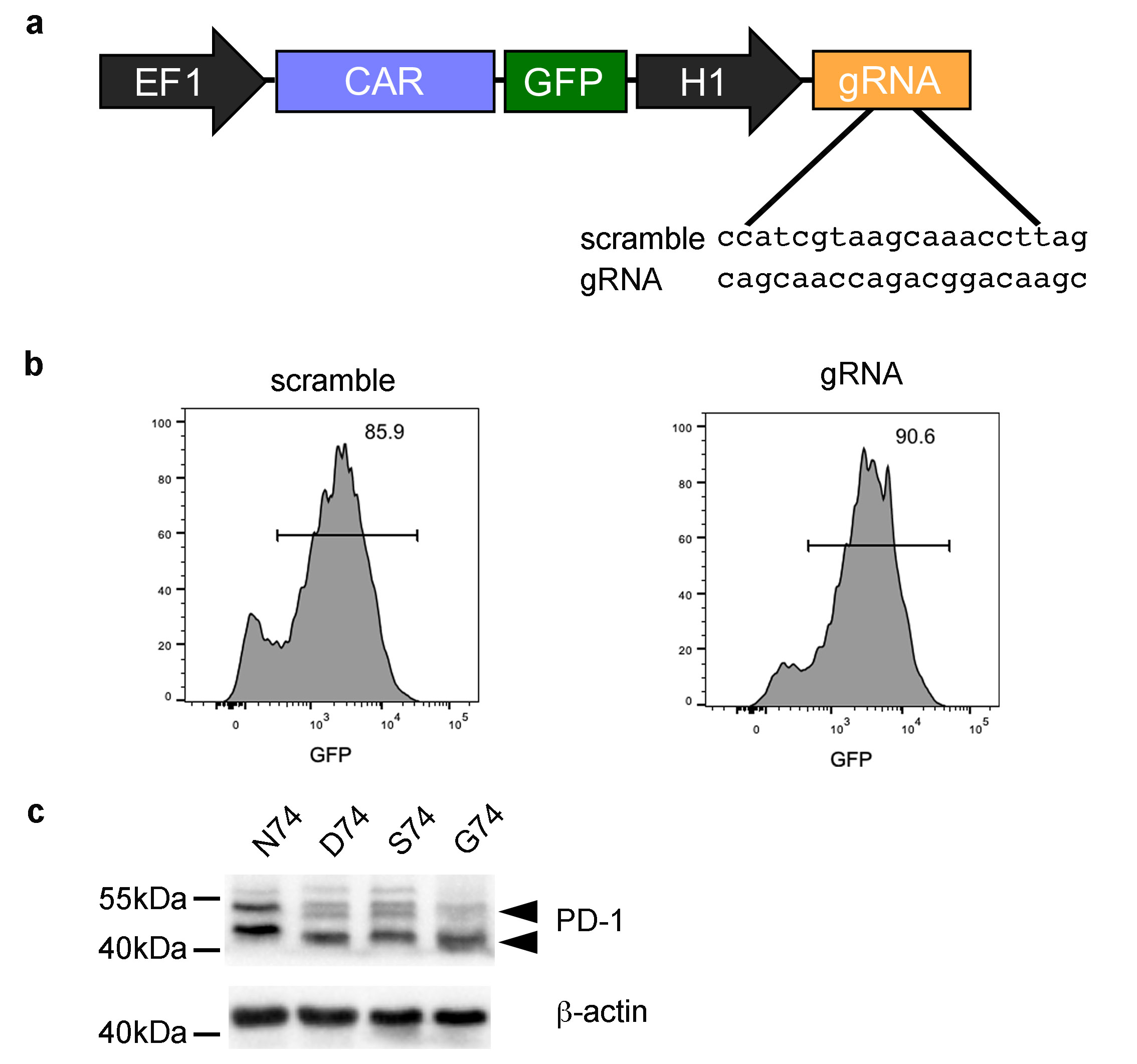

Supplement: Supplementary file 1 — Additional file 1: Figure S1. CAR-T cell construction. (a) Structure of lentiviral vector simultaneously delivering CAR and gRNA. (b) Transduction efficacy of T cells. Transduction efficacies were determined by GFP expression on day 5, before performing single base editing on the same day. (c) Vectors coding wild type (N74) or mutated (D74, S74 or G74) PD-1 were transiently transfected into 293 T cells. 48 hours later, cell lysis was subjected to western blot analysis. This assay showed the alterations at N74 of PDCD1 decreased the expression of PD-1 protein. [file 13045_2019_831_MOESM1_ESM.jpg]

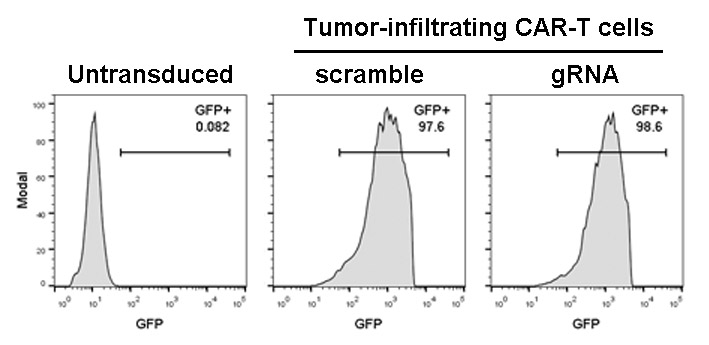

Supplement: Supplementary file 2 — Additional file 2: Figure S2. CAR-T cells divided within tumor. Almost all the T cells (CD45+CD3+) accumulating within tumors were CAR-T cells (GFP+). In the infused T cells, about 85% were GFP+. In the activated T cells within tumors, the ratios of GFP+ cells were over 97%, indicating CAR-T cells but not the non-engineered cells divide upon antigen engagement in vivo. Untransduced T cells were used as control. [file 13045_2019_831_MOESM2_ESM.jpg]
