# Supplementary material for: Targeting glycosylation of PD-1 to enhance CAR-T cell cytotoxicity
Source: J Hematol Oncol. 2019 Nov 29;12:127. doi: 10.1186/s13045-019-0831-5 (PMC6884797; doi:10.1186/s13045-019-0831-5)
Supplement: Supplementary file 3 — Additional file 3: Table S1. Antibodies and materials list. [file 13045_2019_831_MOESM3_ESM.docx]

**Table S1 Antibodies and materials list.**

| Antigen | Catalog # | Clone | Fluorochrome | Vendor |
| --- | --- | --- | --- | --- |
| CD3 | 300316 | HIT3a | PE-Cy7 | BioLegend |
| CD3 | 300310 | HIT3a | PE-Cy5 | BioLegend |
| CD4 | 300514 | RPA-T4 | APC | BioLegend |
| CD4 | 100425 | OKT4 | PE | Capricobio |
| CD4 | 317408 | OKT4 | FITC | BioLegend |
| CD45 | 61-0459-42 | HI30 | eFlour 610 | Invitrogen |
| CD8 | 344714 | SK1 | APC-Cy7 | BioLegend |
| CD69 | 310914 | FN50 | APC-Cy7 | BioLegend |
| CD69 | 310912 | FN50 | PE-Cy7 | BioLegend |
| CD27 | 302808 | O323 | PE | BioLegend |
| CD27 | 356412 | M-T271 | PE-Cy7 | BioLegend |
| PD-1 | 329918 | EH122 | PE-Cy7 | BioLegend |
| PD-1 | 329906 | EH12.2H7 | PE | BioLegend |
| PD-L1 | 374512 | MIH3 | PE | BioLegend |
| PD-1 | ab52587 | NAT105 | n/a | Abcam |
| β-Actin | 58169 | E4D9Z | n/a | CST |
| 7-AAD | 420404 | n/a | PerCP | BioLegend |
| Cell Proliferation Dye | 65-0840-85 | n/a | eFlour 670 | Invitrogen |
| Fixable Viability Dye | 65-0864-14 | n/a | eFluor 660 | Invitrogen |
| D-Luciferin Potassium Salt | 40902es08 | n/a | n/a | Yisheng |
| Human IFN-γ | 300-02-100 | n/a | n/a | PeproTech |

n/a: not applicable.
