# Supplementary material for: Targeting glycosylation of PD-1 to enhance CAR-T cell cytotoxicity
Source: J Hematol Oncol. 2019 Nov 29;12:127. doi: 10.1186/s13045-019-0831-5 (PMC6884797; doi:10.1186/s13045-019-0831-5)
Supplement: Supplementary file 4 — Additional file 4:. Detailed method information and procedures of experiments. [file 13045_2019_831_MOESM4_ESM.docx]

**Materials and Methods**

***Reagents***

Antibodies and materials used in this study can be found in Supplemental Table 1.

***Cell culture***

Tumor cell lines were purchased from Chinese Academy of Sciences Cell Repertoire (Shanghai, China) and were grown in DMEM media supplemented with L-glutamine, 5% fetal bovine serum (FBS) and penicillin/streptomycin (Gibco). T cells were cultured in RPMI-1640 media with 200 IU/mL IL-2 (Peprotech), 5% FBS (Hyclone) and penicillin/streptomycin.

***gRNA design and vector construction***

ABE_max_, PX458 and lentiviral vectors were obtained from Addgene. gRNA targeting the coding sequence of N74 in *PDCD1* or non-specific sequence was designed using web-based tool (<https://sg.idtdna.com/>). The sequences of gRNA were as follow: N74: CAGCAACCAGACGGACAAGC; scramble: CAGCAACCAGACGGACAAGC. Those targeting sequences were synthesized (Sangon Biothech, China) and inserted into PX458 following published protocol (1). To construct stable expression plasmid, gRNA and H1 promoter were inserted into lentiviral vector with mesothelin-targeted CAR coding sequence. CAR was synthesized according to previous report (2).

***ABE editing in T cells***

Firstly, adenine base editor proteins were produced in insect cells according to ABEmax template and purified (Sino Biological, China). Three days after T cell infection with lentivirus, electroporation with ABE proteins was performed according to the protocol adapted from Seki *et al* (3). Briefly, T cells were centrifuged and resuspended in the Lonza electroporation buffer P2 using 100 μL buffer per 5 million cells, supplemented with 200 pmol ABE proteins. Then protocol EH100 was chosen. After electroporation T cells were cultured in complete media with IL-2 (200 IU/mL).

***FACS assessment of T cells***

T cells were mixed with fluorochrome-conjugated antibodies and incubated at 4°C for 30 minutes. After washing with ice-cold PBS with 2% FBS, samples were analyzed with FACSCanto II fluidics system (BD Bioscience). Fixable viability dyes (Thermo Fisher) were added to exclude dead cells. Phenotypes of acquire samples were determined using Flowjo software.

***Preparation of target cells with high PD-L1 expression***

Mesothelin-positive H322 or A549 cells were seeded in advance, followed by IFN-γ (100 IU/mL; Peprotech) stimulation for 24 hours. Then stimulated tumor cells were collected and washed several time with fresh media without exogenous cytokines to discard IFN-γ. Then those PD-L1-expressed tumor cells were used in following experiments.

***Bioluminescence (BLI)-based cytolytic function test of CAR-T cells***

CAR-T cells were adjusted to have comparable GFP^+^ subgroups, washed to discard exogenous cytokines and then incubated with target cells with high PD-L1 at various E:T ratios for 24 hours. Following that, the cytolytic activities of CAR-T cells were determined with IVIS imager (Perkin Elmer).

***ELISA detection of effector cytokines***

CAR-T cells having similar CAR^+^ subsets were washed to discard exogenous cytokines and co-cultured with target cells expressing high levels of PD-L1 at E:T = 1:1 for 24 hours. Following that, IL-2 and IFN-γ in supernatants were tested using specific ELISA kits (RD system).

***Proliferation assay of CAR-T cells***

The ratios of CAR^+^ cells were adjusted to the similar levels and stained with the proliferation-indicative dye eFlour 670 (Thermo Fisher). After washing with fresh media several times, CAR-T cells were co-incubated with target cells at E:T = 1:1 for 48 hours without exogenous cytokines. Then the proliferations of CAR-T cells were determined according to the dilution of eFlour 670.

***Western blot assay***

For protein analysis, cells were lysed in ice-cold lysis buffer (150 mM NaCl, 5 mM EDTA, 50 mM Tris, 1% NP-40, 0.5% sodium deoxycholate, 0.1% SDS) plus protease inhibitor cock- tail (Sigma-Aldrich) and protein concentration was determined using Pierce BCA Protein Assay (Thermo Fisher Scientific). Western blots were performed using 10% SDS-PAGE gel and then transferred onto nitrocellulose transfer membrane using BioRad Blotting System. PD-1 was visualized and quantified using Chemiluminescence Imaging System (Bio-Rad Laboratories).

***RNA isolation and quantitative Real-Time PCR***

RNA was isolated from whole cells using TRIzol reagent (Takara Bio, Japan) according to the manufacturer’s instructions. For cDNA synthesis, 1000 ng total RNA was reverse transcribed using Prime-Script RT reagent Kit with gDNA Eraser (Takara Bio). For quantitative PCR, we used SYBR Green (Roche, German) and on Stratagene Mx3005P qPCR System (Agilent Technologies). Primers for PD-1 are 5’-CAGTTCCAAACCCTGGTGGT-3’ and 5’-GGCTCCTATTGTCCCTCGTG-3’, for GAPDH are 5’-GCACCGTCAAGGCTGAGAAC-3’ and 5’TGGTGAAGACGCCAGTGGA-3’.

***Animal experiments***

Luciferase-expressing H322 cells were inoculated into SCID-beige mice subcutaneously on day -7. On day 0, 1×10^7^ T cells were infused via tail vein when the tumors were visible. In the infused scramble and gRNA cells, the rates (around 85%) of CAR-positive cells were comparable. 5 days after, the tumor-infiltrating CAR-T cells were isolated to check their accumulations, activations and PD-1 expressions. Additionally, tumor growths were monitored weekly after T cell infusion with BLI imager. Once the values of total flux were over 2×10^11^, mice were sacrificed.

***Statistical analysis***

Data are presented as mean ± SD and significance was defined as *P* < 0.05. *T* tests were performed to compare 2 groups. ANOVA tests were carried on to compare the difference among 3 or more groups. All statistical analyses were performed in Graphpad Prism, version 7.0.

**References**

1. Ran FA, Hsu PD, Wright J, Agarwala V, Scott DA, Zhang F. Genome engineering using the CRISPR-Cas9 system. Nat Protoc. 2013;8(11):2281-308.

2. Carpenito C, Milone MC, Hassan R, Simonet JC, Lakhal M, Suhoski MM, et al. Control of large, established tumor xenografts with genetically retargeted human T cells containing CD28 and CD137 domains. Proc Natl Acad Sci U S A. 2009;106(9):3360-5.

3. Seki A, Rutz S. Optimized RNP transfection for highly efficient CRISPR/Cas9-mediated gene knockout in primary T cells. J Exp Med. 2018;215(3):985-997.
